# Terazosin as a Non-Hormonal Treatment for Endometriosis

**DOI:** 10.3390/ijms27094093

**Published:** 2026-05-02

**Authors:** Ahmet Beyazıt, Okan Tutuk, Didar Gürsoy Kuzuluk

**Affiliations:** 1 Department of Obstetrics and Gynecology, Hatay Mustafa Kemal University, Antakya 31060, Hatay, Türkiye; 2 Department of Physiology, Hatay Mustafa Kemal University, Antakya 31060, Hatay, Türkiye; 3 Department of Pathology, Hatay Mustafa Kemal University, Antakya 31060, Hatay, Türkiye

**Keywords:** endometriosis, terazosin, steroidogenic factor-1, inflammation, rat model

## Abstract

Endometriosis is a chronic, estrogen-dependent inflammatory disease including aberrant local steroidogenesis, inflammation, angiogenesis, oxidative stress, and prostaglandin-mediated pain. Given the elevated adrenergic receptor expression in endometriotic lesions and the potential of terazosin to downregulate Steroidogenic Factor-1 (SF-1), this study aimed to evaluate terazosin as a non-hormonal therapy in a surgically induced rat endometriosis model. Forty female Wistar rats were randomized to sham, untreated endometriosis, leuprolide acetate or terazosin; two postoperative deaths yielded final group sizes of 10/9/10/9. Blinded histopathology verified successful lesion establishment. ELISA quantified SF-1, IL-6, IL-8, TNF-α, NF-κB, VEGF, HIF-1α, and PGE2 in lesion tissue, serum, and peritoneal lavage; oxidative status was assessed by TAS, TOS, and OSI. Compared with untreated endometriosis, terazosin significantly reduced SF-1, PGE2, IL-6, IL-8, TNF-α, VEGF and HIF-1α across compartments (all *p* < 0.001), comparable to leuprolide (*p* = 1.000). Terazosin also normalized oxidative stress by decreasing TOS/OSI and restoring TAS in tissue, serum, and peritoneal fluid (*p* < 0.001). NF-κB decreased in tissue and serum (*p* < 0.001) but not in peritoneal fluid (*p* = 0.206). Overall, terazosin produced leuprolide-like molecular benefits without hormonal suppression, supporting repurposing as a candidate non-hormonal therapy, while highlighting the need for longer-duration studies and randomized clinical trials given model and pain-assessment limitations.

## 1. Introduction

Endometriosis, affecting over 176 million women globally, is a chronic inflammatory condition with a prevalence of approximately 10% among women of reproductive age, and it develops in an estrogen-dependent manner [1,2]. This disorder is characterized by the presence of endometrial-like tissue outside the uterine cavity, significantly impairing patients’ quality of life through symptoms such as chronic pelvic pain, dysmenorrhea, dyspareunia, and infertility [3].

**Figure 1:**
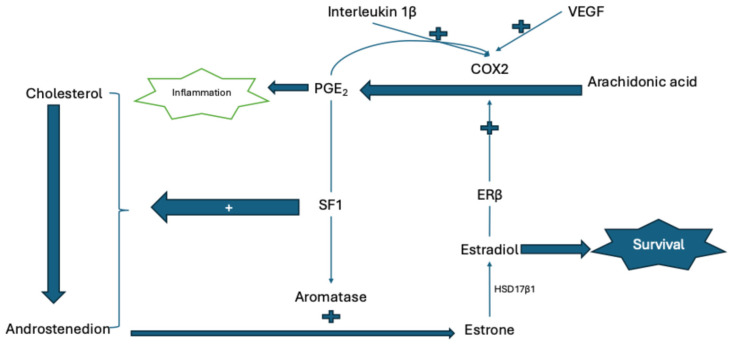
Schematic representation of the cellular and molecular pathophysiology of endometriosis. HSD17β1: 17β-hydroxysteroid dehydrogenase type 1; ERβ: Estrogen receptor beta; COX-2: Cyclooxygenase-2; VEGF: Vascular endothelial growth factor; PGE2: Prostaglandin E2; SF-1: Steroidogenic factor-1.

While the pathophysiology of the disease remains incompletely understood, it is hypothesized that various mechanisms, including immunological dysfunction, abnormal endocrine signaling, angiogenesis, neurogenic inflammation, and oxidative stress, contribute to its development [4]. Estrogen dominance is pivotal in the disease’s pathogenesis. In endometriosis implants, the presence of aromatase, which synthesizes estrone from androgens, and 17-hydroxysteroid dehydrogenase Type 1 (HSD17β1), which converts estrone to estradiol, is noted, whereas Type 2 is absent. Type 2 typically maintains hormonal balance by converting estradiol back to estrone; however, its absence in endometriosis implants results in the continuous synthesis of estradiol from estrone. Consequently, estradiol, a more potent estrogen than estrone, becomes predominant in endometrial implants. Elevated estradiol levels, in conjunction with interleukins and cytokines, activate the cyclooxygenase-2 (COX2) enzyme. COX2, through the activation of the prostaglandin synthetase enzyme, synthesizes prostaglandin E2 (PGE2) from arachidonic acid, a mechanism responsible for pain. The synthesized PGE2 further activates the aromatase enzyme and steroidogenesis. Aromatases also catalyze the synthesis of estrone from androstenedione and estradiol via 17-hydroxysteroids. This process perpetuates a vicious cycle, resulting in an excessively estrogenic environment [5] (Figure 1).

The management of endometriosis involves medical and surgical interventions. Medical treatment includes hormonal therapies for symptom management and lesion suppression. Treatment primarily focuses on suppressing estrogen dominance at ectopic sites through pharmacological agents targeting the steroidogenic pathway [6]. The principal strategy in treating endometriosis focuses on suppressing estrogen dominance at ectopic sites. To achieve this, various pharmacological agents targeting distinct steps in the steroidogenic pathway are employed. Among the primary options are combined oral contraceptives to inhibit HSD17β1 activity, progestins to counteract estrogenic effects, non-steroidal anti-inflammatory drugs (NSAIDs) to suppress the pro-inflammatory process and COX enzyme, and aromatase inhibitors to limit peripheral aromatase activity. Although GnRH analogs, which suppress the hypothalamic-pituitary-ovarian axis, are regarded as some of the most potent agents in medical treatment, their associated side effects, such as severe hypoestrogenism and loss of bone mineral density, present significant challenges to their long-term use [7]. Due to these limitations, along with the contraceptive nature of conventional hormonal therapies, there is a critical need for alternative, non-hormonal treatments. Consequently, various novel therapeutic approaches targeting angiogenesis, neuroinflammation, immune dysfunction, and specific non-hormonal molecular pathways are currently being extensively investigated [2,8,9].

Terazosin, a long-acting α-1 adrenergic receptor antagonist, received initial approval from the FDA in 1987 for the management of hypertension and subsequently in 1993 for benign prostatic hyperplasia. This pharmacological agent is generally well tolerated, with its side effect profile primarily attributable to the blockade of α-1 receptors. The most frequently observed adverse effects include dizziness, headache, fatigue, nasal congestion, and orthostatic hypotension. Terazosin has been safely utilized by patients over an extended period [10]. Given the elevated expression of adrenergic receptors identified in endometriotic lesions [11] and its potential downregulatory effects on Steroidogenic Factor-1 (SF-1), we hypothesize that terazosin may present a novel and efficacious therapeutic strategy for alleviating endometriosis symptoms. To assess the validity of this hypothesis, we investigated the efficacy of terazosin administration in a surgically induced endometriosis rat model, employing both molecular and histopathological parameters.

## 2. Results

A total of 40 rats were initially included in the experimental design. During the experimental period, two postoperative mortalities occurred following the second laparotomy due to surgical stress and anesthetic complications (one rat in the Endometriosis control group and one rat in the Terazosin-treated group). Consequently, the study was successfully completed with 38 rats (sham: *n* = 10; endometriosis: *n* = 9; terazosin: *n* = 9; leuprolide: *n* = 10). The results of the enzyme-linked immunosorbent assay (ELISA) analyses performed are given in Table 1, Table 2 and Table 3.

In the histopathological analysis conducted on all rats with surgically induced endometriosis, it was verified that the model was successfully established, with the exception of the Sham group.

**Figure 2:**
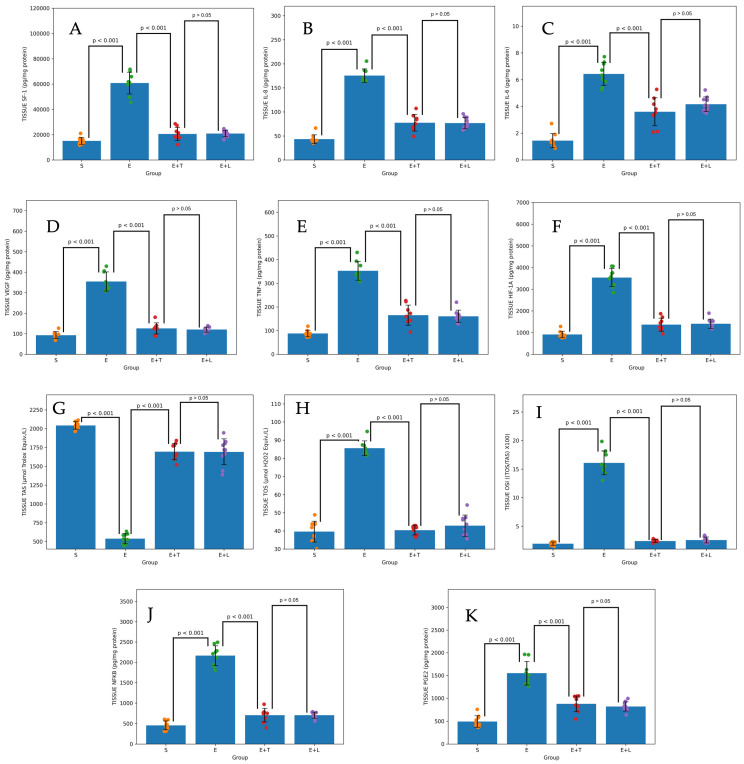
Effects of terazosin and leuprolide acetate treatments on molecular and biochemical parameters in endometriotic tissue. Bar graphs illustrate the tissue concentrations of (**A**) Steroidogenic Factor-1 (SF-1), (**B**) Interleukin-8 (IL-8), (**C**) Interleukin-6 (IL-6), (**D**) Vascular Endothelial Growth Factor (VEGF), (**E**) Tumor Necrosis Factor-alpha (TNF-α), (**F**) Hypoxia-Inducible Factor-1 Alpha (HIF-1A), (**G**) Total Antioxidant Status (TAS), (**H**) Total Oxidant Status (TOS), (**I**) Oxidative Stress Index (OSI), (**J**) Nuclear Factor kappa-B (NF-κB), and (**K**) Prostaglandin E2 (PGE2). S: Sham group; E: Endometriosis control group.

**Figure 3:**
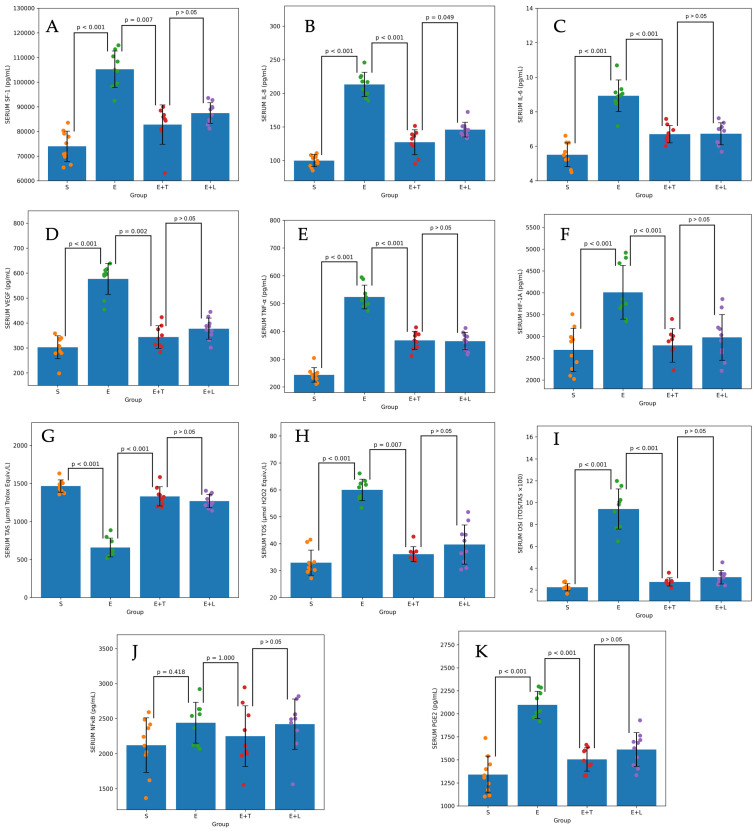
Effects of terazosin and leuprolide acetate treatments on molecular and biochemical parameters in serum. Bar graphs illustrate the tissue concentrations of (**A**) Steroidogenic Factor-1 (SF-1), (**B**) Interleukin-8 (IL-8), (**C**) Interleukin-6 (IL-6), (**D**) Vascular Endothelial Growth Factor (VEGF), (**E**) Tumor Necrosis Factor-alpha (TNF-α), (**F**) Hypoxia-Inducible Factor-1 Alpha (HIF-1A), (**G**) Total Antioxidant Status (TAS), (**H**) Total Oxidant Status (TOS), (**I**) Oxidative Stress Index (OSI), (**J**) Nuclear Factor kappa-B (NF-κB), and (**K**) Prostaglandin E2 (PGE2). S: Sham group; E: Endometriosis control group; E + T: Endometriosis + Terazosin treatment group; E + L: Endometriosis + Leuprolide acetate treatment group.

**Figure 4:**
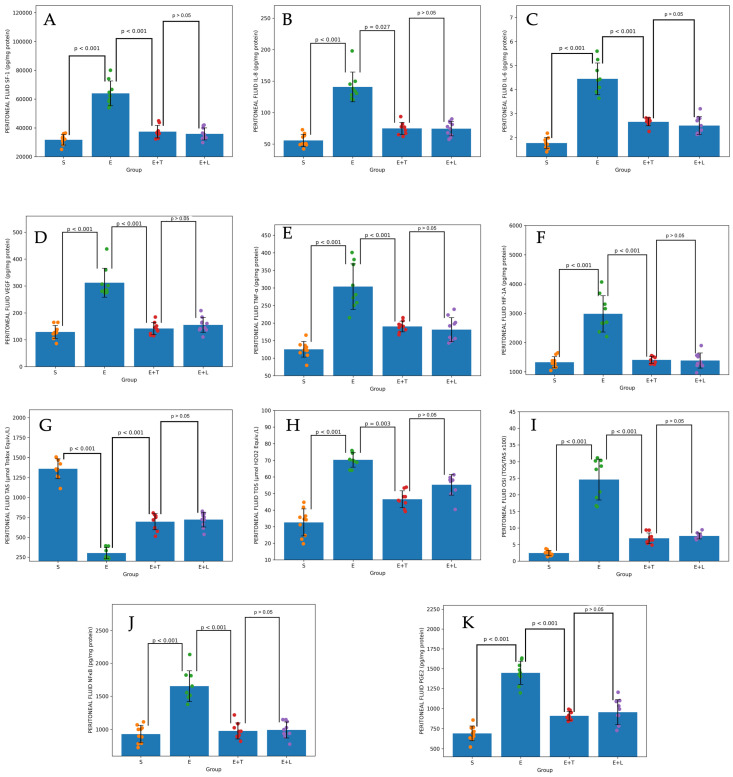
Effects of terazosin and leuprolide acetate treatments on molecular and biochemical parameters in peritoneal fluid. Bar graphs illustrate the tissue concentrations of (**A**) Steroidogenic Factor-1 (SF-1), (**B**) Interleukin-8 (IL-8), (**C**) Interleukin-6 (IL-6), (**D**) Vascular Endothelial Growth Factor (VEGF), (**E**) Tumor Necrosis Factor-alpha (TNF-α), (**F**) Hypoxia-Inducible Factor-1 Alpha (HIF-1A), (**G**) Total Antioxidant Status (TAS), (**H**) Total Oxidant Status (TOS), (**I**) Oxidative Stress Index (OSI), (**J**) Nuclear Factor kappa-B (NF-κB), and (**K**) Prostaglandin E2 (PGE2). S: Sham group; E: Endometriosis control group; E + T: Endometriosis + Terazosin treatment group; E + L: Endometriosis + Leuprolide acetate treatment group.

Upon examining the levels of SF-1, a principal regulator of local estrogen production in endometriotic tissues, a significant elevation was noted in the Endometriosis group compared to the Sham group (*p* < 0.001). Terazosin treatment markedly suppressed tissue SF-1 expression relative to the Endometriosis group (*p* < 0.001). This suppressive effect was statistically comparable to the reduction observed in the leuprolide group (*p* >0.05) (Figure 2A). A similar pattern was also observed in peritoneal and serum samples (Figure 3A and Figure 4A).

The highest concentrations of pro-inflammatory cytokines, specifically interleukin-6 (IL-6), interleukin-8 (IL-8), and tumor necrosis factor-alpha (TNF-α), as well as the transcription factor nuclear factor kappa B (NF-κB), which are implicated in disease progression, were observed in group E. In the cohort treated with terazosin, tissue levels of IL-6, IL-8, and TNF-α were significantly diminished in comparison to the control group (for all, *p* < 0.001) (Figure 2B,C,E). This significant reduction was also evident in peritoneal and serum samples (Figure 3B,C,E and Figure 4B,C,E). Although a significant difference in NF-κB levels was detected in tissue and serum (*p* < 0.001), this difference was not statistically significant in peritoneal fluid (*p* = 0.206) (Figure 2J, Figure 3J and Figure 4J).

Similarly, it was observed that the levels of vascular endothelial growth factor (VEGF) and hypoxia-inducible factor 1-alpha (HIF-1 α), which serve as indicators of angiogenesis and hypoxia, were also reduced by terazosin treatment, demonstrating an efficacy comparable to that of the leuprolide group (*p* >0.05) (Figure 2D–F, Figure 3D–F and Figure 4D–F).

In the endometriosis control group, a pronounced oxidative stress profile was confirmed by the observation of elevated oxidative stress index (OSI) and total oxidant status (TOS) levels, accompanied by a reduction in total antioxidant status (TAS). Administration of terazosin resulted in a significant decrease in TOS and OSI values (*p* < 0.001) and restored antioxidant capacity (TAS) in tissue, serum, and peritoneal fluid, aligning it more closely with physiological levels observed in the Sham group (Figure 2G–I, Figure 3G–I and Figure 4G–I).

In assessing the concentrations of PGE2, a principal mediator of pain in endometriosis, it was found that terazosin treatment significantly decreased PGE2 accumulation in ectopic tissues compared to the Endometriosis group (*p* < 0.001). This reduction in PGE2 levels was statistically comparable to that observed in the group receiving leuprolide treatment, which induces systemic estrogen suppression (*p* >0.05) (Figure 2K). A similar statistical relationship was observed in serum and peritoneal fluid samples (Figure 3K and Figure 4K).

Furthermore, effect sizes (r) were calculated for the non-significant pairwise comparisons between the Terazosin (E + T) and Leuprolide (E + L) groups. Across the evaluated parameters, the effect sizes were found to be small in serum (r = 0.01–0.32; mean r = 0.18), peritoneal fluid (r = 0.01–0.33; mean r = 0.14), and tissue samples (r = 0.02–0.18; mean r = 0.08) (Table 1, Table 2 and Table 3).

Statistically significant differences were observed among the groups’ histochemical staining scores (*p* < 0.001). The results of the Dunn–Bonferroni post hoc analysis for pairwise comparisons revealed that the histochemical scores in the untreated endometriosis group were significantly higher than those in the sham and both treatment groups (*p* < 0.05 for all comparisons). When comparing the Terazosin (E + T) and Leuprolide (E + L) treatment groups, no statistically significant difference was detected in terms of histopathological regression (*p* > 0.05). Similarly, the absence of a significant difference between the treatment groups and the sham group (*p* > 0.05) suggests that the applied treatments brought the morphological structure closer to healthy tissue levels (Table 4).

## 3. Discussion

Endometriosis is a chronic, estrogen-dependent inflammatory disorder affecting women of reproductive age, characterized by the presence of endometrial glands and stroma outside the uterine cavity. Its etiopathogenesis is notably complex; increased local steroidogenesis, prompted by cellular-level abnormalities, elevated oxidative stress, pro-inflammatory cytokine activation, aberrant angiogenesis, and neurogenic pain mediators, constitute the primary pathophysiological cascades facilitating the survival and progression of ectopic lesions [12]. The side effect profiles and usage limitations of current medical treatments necessitate the exploration of novel pharmacological agents capable of simultaneously targeting these multiple mechanisms.

In this context, we investigated the therapeutic efficacy of terazosin, an α-1 adrenergic receptor antagonist, in a surgically induced rat model of endometriosis. The principal finding of the study is that terazosin administration significantly suppressed, the parameters of oxidative stress, inflammation, angiogenesis, and pain—key factors in the disease’s progression—as evidenced in tissue samples as well as in serum and peritoneal fluid specimens. Notably, this multifaceted pathophysiological suppression achieved by terazosin was found to be comparable to that of leuprolide which is regarded as one of the most potent agents in clinical practice. The quantitative analysis of our findings substantiates the clinical comparability, as the effect sizes between the two treatment groups consistently remained small across all assessed microenvironments. These results suggest that terazosin could serve as a potent, non-hormonal, and multifunctional therapeutic alternative in the management of endometriosis.

In the pathophysiology of endometriosis, SF-1 serves as a pivotal transcription factor that governs local estrogen production within ectopic tissues. In a healthy endometrium, the SF-1 promoter region is typically suppressed due to extensive methylation. However, in endometriotic stromal cells, epigenetic hypomethylation results in the aberrant expression of this gene [12,13]. SF-1 concurrently activates the aromatase and Steroidogenic Acute Regulatory Protein (StAR) genes, facilitating the conversion of cholesterol to estradiol. The increased local estradiol subsequently stimulates the COX-2 enzyme, resulting in elevated PGE2 synthesis. The rising levels of PGE2 then activate the cyclic adenosine monophosphate (cAMP) pathway, which in turn reactivates SF-1. This deleterious positive feedback loop (SF-1/Aromatase/PGE2) constitutes the fundamental mechanism that ensures the persistence of the lesion and perpetuates the inflammatory process [14].

Previous research has demonstrated that terazosin, an α-1 adrenergic receptor antagonist, mitigates androgen synthesis and oxidative stress by downregulating the SF-1 signaling pathway in LH-induced bovine theca cells [15]. In this study, informed by these mechanistic insights, we observed that SF-1 expression in the terazosin-treated cohort was significantly lower than in the untreated cohort (*p* < 0.001). Importantly, no statistically significant difference was detected in SF-1 levels between the effects of terazosin and leuprolide (*p* > 0.05). These results indicate that terazosin is as efficacious as leuprolide in disrupting the epigenetically driven steroidogenic cycle at ectopic sites. Furthermore, given its acceptable side effect profile compared to GnRH analogs—whose use is constrained by hypoestrogenic side effects—terazosin may represent a viable therapeutic alternative.

### 3.1. Oxidative Stress

In the pathophysiology of endometriosis, the disequilibrium between reactive oxygen species (ROS) and antioxidant defense mechanisms is pivotal in initiating inflammation, angiogenesis, and ectopic tissue proliferation [16]. The literature indicates that elevated oxidative stress levels enhance the expression of pro-inflammatory cytokines via NF-κB activation and facilitate lesion survival by promoting vascularization through VEGF. Notably, lipid peroxidation and resistance to ferroptosis, resulting from the accumulation of free iron in the peritoneal cavity due to retrograde menstruation, have been identified as critical processes in the disease’s progression [17,18,19,20,21].

The biochemical analysis results presented in this study align comprehensively with the mechanisms delineated in the existing literature. The observation that TOS and OSI levels were significantly elevated in the endometriosis group compared to the sham group (*p* < 0.001) indicates that our model accurately represents the oxidative damage cycle. Notably, the administration of terazosin resulted in a statistically significant reduction in this oxidative burden (both TOS and OSI levels) (*p* < 0.001). The finding that the antioxidant effect of terazosin was comparable to that of leuprolide acetate—known for its high efficacy in treating endometriosis (*p* > 0.05) enhances the therapeutic potential of terazosin. In conclusion, our data demonstrate that, in addition to downregulating SF-1, terazosin effectively modulates oxidative stress pathways, thereby mitigating the development of endometriotic foci.

### 3.2. Pain

Neurogenic inflammation plays a pivotal role in the pathophysiology of chronic pelvic pain associated with endometriosis. Within this context, Nerve Growth Factor (NGF) is identified as a crucial mediator that exacerbates pain severity by facilitating the proliferation, sensitization, and neurogenesis of nerve fibers [22,23]. Similarly, PGE2, synthesized via the COX-2 enzyme, is recognized as one of the most potent molecules that initiates both the inflammatory response and nociceptive transmission [24].

Current therapeutic approaches employ NSAIDs to provide symptomatic relief through COX inhibition, while progestins such as dienogest suppress NGF and pro-inflammatory cytokine expression [24,25,26]. However, the limitations associated with the long-term use of these agents underscore the need for novel non-hormonal strategies targeting the pain pathway. In this study, the administration of terazosin demonstrated a significant suppressive effect on the molecular markers of endometriosis-related pain. The efficacy of terazosin was found to be comparable to that of leuprolide, suggesting its potential to effectively modulate neurogenic inflammation and pain sensitization at ectopic sites.

### 3.3. Inflammatory Response

The chronic inflammatory process central to the pathogenesis of endometriosis is characterized by elevated levels of pro-inflammatory cytokines, including TNF-α, IL-6, and IL-8, in both the peritoneal fluid and lesion sites. These elevated cytokine levels directly induce the proliferation, adhesion, and angiogenesis of ectopic cells, thereby perpetuating the progression of the disease [27,28]. Recent studies have indicated that progestins, such as dienogest, and GnRH agonists, such as goserelin, achieve therapeutic efficacy by markedly reducing cytokine levels [29,30,31,32]. Nonetheless, it has been documented that the impact of surgical excision on cytokine levels is transient, with levels beginning to increase again by the third postoperative month [33]. This underscores the necessity for the development of novel pharmacological agents that demonstrate long-term efficacy and reduced adverse effects in order to effectively suppress the inflammatory cascade.

The findings of our study indicate that terazosin exhibits significant potential in modulating the inflammatory response associated with endometriosis. Specifically, for the three pro-inflammatory cytokines—TNF-α, IL-6, and IL-8—terazosin was observed to significantly reduce levels in tissue, serum, and peritoneal fluid within the endometriosis cohort (*p* < 0.001). This effect was comparable to that of leuprolide acetate (*p* > 0.05). These results suggest that terazosin may serve as a potent alternative or adjunctive treatment to hormonal and surgical therapies in the management of endometriosis.

Notably, although terazosin significantly reduced NF-κB levels in both endometriotic tissue and peritoneal fluid, no statistically significant difference was observed in systemic serum NF-κB concentrations among the groups. This discrepancy is likely attributable to the pathophysiological characteristics of endometriosis, which is primarily driven by a localized inflammatory microenvironment rather than a generalized systemic immune response. The ectopic endometrial implants and the surrounding peritoneal fluid serve as the primary epicenter for cytokine production and NF-κB activation.

### 3.4. Angiogenesis

Angiogenesis is a critical factor in the survival and invasive potential of endometriotic lesions, as it relies on the establishment of a robust vascular network surrounding the lesion. The primary mediator of this process, VEGF, is intricately regulated by pro-inflammatory cytokines, such as HIF-1α, and local estrogen levels [34]. The literature indicates that, in addition to anti-angiogenic agents like bevacizumab, hormonal therapies such as dienogest and danazol contribute to lesion regression by inhibiting VEGF expression [29,35,36]. Furthermore, surgical excision has been shown to significantly decrease serum VEGF levels by ameliorating the angiogenic milieu [37]. However, the invasive nature of surgical interventions and the adverse effect profile of hormonal therapies underscore the necessity for non-hormonal pharmacological alternatives that specifically target angiogenesis.

In this study, we observed that the administration of terazosin exerted a significant suppressive effect on VEGF levels within endometriotic foci, comparable to the effects of leuprolide. We posit that attenuation associated with terazosin’s downregulatory effect on SF-1, coupled with its reduction in oxidative stress, may disrupt the vascular support of the lesion by diminishing the biochemical stimuli essential for angiogenesis. Our data suggest that terazosin effectively modulates the angiogenic cascade and suppresses the progression of ectopic tissues.

### 3.5. Histopathological Evaluation

To corroborate our biochemical findings with morphological evidence, histochemical analyses of endometriosis lesions were conducted. As anticipated, significant morphological impairments were observed in the untreated group, whereas terazosin treatment markedly reversed this histopathological progression. Notably, one of the most significant findings of our study is that the tissue-level improvement with terazosin was clinically comparable to that observed in the standard leuprolide treatment groups. The absence of a statistical difference between these two treatments (*p* > 0.05) provides robust quantitative evidence that both drugs exhibit similar efficacy. This morphological regression indicates that the therapeutic effect of terazosin not only involves the suppression of biochemical mediators but also achieves a physical reduction in ectopic lesions at a level comparable to standard hormone therapy.

In clinical practice, it is well known that there is not always a linear correlation between the extent of histopathological lesions in endometriosis and the severity of the clinical picture; relatively small lesions can lead to severe and debilitating pain, while morphologically widespread disease may present with mild or asymptomatic clinical profiles [8,38]. Interestingly, in the experimental in vivo model we designed, it was observed that the morphological regression of ectopic lesions was highly simultaneous and consistent with the suppression of biochemical and molecular parameters. This synchronized improvement seen both in tissue architecture and in molecular inflammatory cascades indicates that terazosin exerts a comprehensive therapeutic effect that simultaneously targets both the macroscopic progression of the disease and its underlying biochemical triggers.

Although our findings demonstrate a significant reduction in various pathological markers, a limitation of the current study is the interdependent nature of the measured mediators (e.g., cytokines, VEGF, and SF-1). Because these pathways are highly interconnected, it is challenging to definitively distinguish the direct molecular targets of terazosin from its indirect downstream anti-inflammatory effects. The hypothesis regarding the modulation of SF-1 by terazosin in our endometriosis model builds upon previous in vitro evidence demonstrating that terazosin specifically reduces SF-1 expression in bovine ovarian theca cells [15]. While our in vivo results strongly align with these cellular findings and indicate a significant downregulation of the SF-1 pathway, our current data remain primarily correlative. Therefore, the exact mechanism should be interpreted with caution, and further molecular binding studies are required to establish whether this represents a direct inhibition within endometriotic lesions or an indirect consequence of generalized disease regression.

A critical advantage of exploring terazosin as an alternative therapeutic agent for endometriosis lies in its non-hormonal mechanism of action. Current medical mainstays, such as the GnRH agonist leuprolide used in our positive control group, exert their effects by inducing a severe hypoestrogenic state [6]. While effective for lesion regression, this mechanism inherently leads to debilitating side effects, most notably bone mineral density loss and severe vasomotor symptoms. Furthermore, because these hormonal suppressants inhibit ovulation, they are fundamentally contraindicated for endometriosis patients actively seeking to conceive [39]. In contrast, terazosin modulates disease progression through targeted mechanistic pathways without disrupting the hypothalamic-pituitary-gonadal axis. This non-contraceptive nature makes it a highly promising therapeutic alternative for women desiring pregnancy. While direct evaluation of systemic side effects was beyond the scope of this short-term study, terazosin theoretically circumvents the severe hypoestrogenism associated with leuprolide. Although terazosin may cause mild alpha-adrenergic side effects, such as orthostatic hypotension or dizziness, it is an FDA-approved medication with a well-established, highly reliable clinical safety profile spanning decades. Future longitudinal studies are warranted to clinically validate this comparative safety and fertility-sparing advantage.

### 3.6. Limitations

The present study acknowledges several limitations. Firstly, the research employed an experimental rat model, which necessitates caution when extrapolating the findings to human clinical contexts. The physiological differences between the estrous cycle of rats and the human menstrual cycle, along with the fact that endometriosis does not spontaneously develop in these animals, are significant considerations. Secondly, the 14-day treatment period offers a limited timeframe to assess the long-term efficacy of the drug in managing chronic endometriosis and its potential late side effects. While this design was robustly suited for our proof-of-concept objective to establish the presence of a therapeutic effect, the lack of a detailed dose–response evaluation limits the broader generalizability of our findings. Therefore, the results presented herein should be considered preliminary and hypothesis-generating. Future comprehensive studies incorporating various dose–response combinations and longitudinal time-dependent assessments are essential to fully elucidate the therapeutic window and long-term efficacy of terazosin in endometriosis. Lastly, the analysis of pain parameters solely through biochemical mediators may not fully capture the complexity of clinical pain perception. Consequently, the absence of functional behavioral pain assays (such as von Frey or writhing tests) to corroborate actual analgesic efficacy represents a notable limitation of the present study. Nonetheless, this study provides an original contribution to the literature, as it is the first to comprehensively elucidate the therapeutic potential of terazosin at the molecular level.

## 4. Materials and Methods

**Figure 5:**
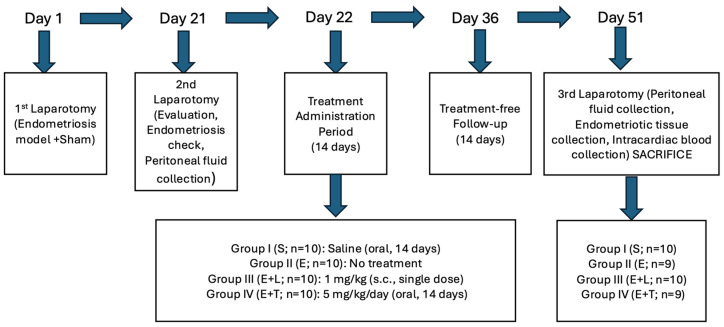
Comprehensive experimental workflow and timeline. The schematic represents the sequentially planned procedures, including the three laparotomies, induction of endometriosis model, confirmatory assessment of lesions, grouping, and specific treatment durations. Abbreviations: s.c., subcutaneous.

The study protocol was approved by the Hatay Mustafa Kemal University Animal Experiments Local Ethics Committee (Date: 27 February 2025, Decision No: 2025/02-09). All experimental procedures were performed in accordance with the ethical guidelines for the care and use of laboratory animals. The study protocol is comprehensively outlined in Figure 5.

The determination of the number of animals required for the study adhered to the “3R” (Replacement, Reduction, Refinement) ethical principle, which is essential in the utilization of experimental animals. Initially, the minimum sample size was calculated to necessitate a total of 52 subjects, with 13 allocated to each group. This estimation was based on the following parameters: a Type I error rate (α) of 0.05, a targeted statistical power (1 − β) of 80%, and an effect size (f) of 0.40 (approximately equivalent to Cohen’s d ≈ 0.8). However, given the anticipated substantial therapeutic effect size of terazosin application (Cohen’s d ≥ 1.0), an optimization was conducted. Considering potential attrition of animals during the experiment, it was determined that 10 animals per group (a total of 40 animals) would suffice to maintain statistical significance and 80% power, representing the scientific minimum required.

### 4.1. Animal Groups and Experimental Design

The study utilized forty adult female Wistar albino rats, aged 6–8 weeks and weighing 250 ± 50 g. These subjects were randomly allocated into four groups, each comprising ten rats (*n* = 10). The animals were maintained in an environment with controlled temperature (24 ± 2 °C) and humidity (50–60%), under a 12 h light/dark cycle, with unrestricted access to a standard diet and water. According to the experimental protocol, Group I (S) underwent a sham operation, while Group II (E) served as the untreated endometriosis model. Group III (E + L) was administered leuprolide (1 mg/kg, s.c., single dose), recognized for its therapeutic efficacy. Group IV (E + T) received terazosin (5 mg/kg/day, oral gavage, for 14 days), which was the primary focus of the research. Terazosin was procured in its commercial tablet form (Hytrin^®^). For administration purposes, the tablets were meticulously pulverized using a sterile mortar and pestle, followed by dissolution or suspension in a 0.9% sterile saline solution to achieve the required concentration. The solution was freshly prepared each day prior to administration. The dosages and routes of administration for both therapeutic agents were selected based on established studies demonstrating their efficacy in rat models [40,41].

### 4.2. Surgical Procedures

During all surgical interventions, the rats were administered intramuscular general anesthesia, comprising 80 mg/kg ketamine (Ketalar; Eczacibasi, Turkey) and 12 mg/kg xylazine (Rompun; Bayer Sisli, Turkey). The depth of anesthesia was maintained with supplementary doses as required. Following the shaving of the surgical sites, antisepsis was achieved using 10% povidone-iodine. Access to the abdominal cavity was obtained via a median incision measuring approximately 3–4 cm.

#### 4.2.1. 1st Laparotomy: Establishment of the Endometriosis Model

The experimental model of endometriosis was developed by modifying the autotransplantation technique as described by Rajkumar [42]. One horn of the rat uterus was ligated at the cervical and utero-ovarian junctions and subsequently resected. The excised uterine tissue was longitudinally opened in sterile saline and sectioned into 5 mm fragments. These endometrial tissues were then implanted onto the peritoneal wall using 5-0 Vicryl sutures. In the sham group, only laparotomy was performed without tissue transplantation.

#### 4.2.2. 2nd Laparotomy: Model Confirmation and Treatment Initiation

Twenty-one days post-induction surgery, a second laparotomy was conducted to confirm the development of endometriotic foci. The abdomen was closed in accordance with anatomical layers. In the postoperative period, the following protocols were applied according to the groups:

- Group S (Sham): Oral gavage with saline solution for 14 days.
- Group E (Endometriosis): Monitored without treatment.
- Group E + T (Terazosin): Oral gavage with terazosin by dissolving the tablets in sterile saline at a daily dose of 5 mg/kg (Hytrin^®^, 5 mg; Abbott, Türkiye) for 14 days.
- Group E + L (Positive Control): A single subcutaneous dose of leuprolide (Lucrin Depot^®^, Abbott, Istanbul, Türkiye) (1 mg/kg).

#### 4.2.3. 3rd Laparotomy: Sampling and Sacrificing

**Figure 6:**
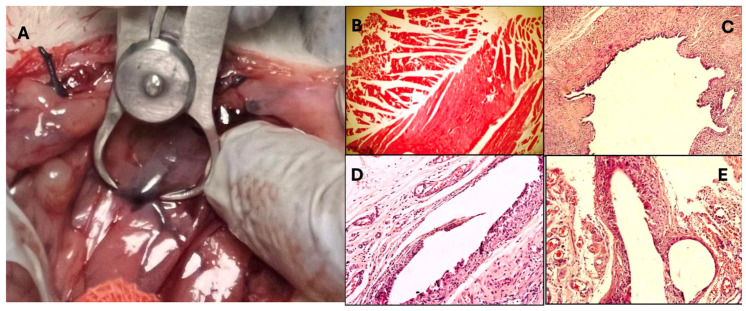
Macroscopic and microscopic evaluation of endometriotic lesions. (**A**) Macroscopic appearance of an autotransplanted endometrial explant on the rat peritoneum during the third laparotomy. (**B**–**E**) Representative photomicrographs of hematoxylin and eosin (H&E)-stained sections of endometriotic foci from different study groups. (**B**) No epithelium (score 0) in sham group. (**C**) A well-preserved epithelial layer (score3) in endometriosis group. (**D**) A moderately preserved epithelium (score 2) in E + T group. (**E**) A poorly preserved epithelium (score1) in E + L group.

At the conclusion of the treatment and follow-up period (on day 51 of the experiment), a terminal laparotomy was conducted on the subjects. Following the collection of peritoneal lavage fluid samples, endometriotic lesions were excised for subsequent analysis. Upon completion of the procedure, whole blood samples were obtained via intracardiac blood sampling, and the rats were euthanized (Figure 6).

### 4.3. Histopathological Evaluation

Following fixation in a 10% buffered formalin solution, the excised endometriotic tissue samples underwent standard tissue processing procedures and were subsequently embedded in paraffin blocks. Sections were cut to a thickness of 5 μm and stained with Hematoxylin-Eosin (H&E). A blinded pathologist assessed the morphological alterations and epithelial integrity of the endometriotic lesions using a light microscope, employing the semi-quantitative scoring system delineated by Keenan et al. [43]. According to this system, lesions were categorized as follows: a well-preserved epithelial layer = score 3, a moderately preserved epithelium = score 2, a poorly preserved epithelium (occasional epithelial cells only) = score 1, and no epithelium = score 0. The absence of epithelium (Score 0) was defined as such (Figure 6).

### 4.4. Biochemical and Molecular Analyses

#### 4.4.1. Tissue Homogenization and Protein Quantification

Excised endometriotic implants and normal tissues were immediately rinsed with ice-cold phosphate-buffered saline (PBS) to remove excess blood. Tissue samples were homogenized in ice-cold PBS using a tissue homogenizer and then centrifuged at 10,000× *g* for 15 min 4 °C. The clear supernatants were carefully collected and stored at −80 °C until further analysis. To normalize the biomarker concentrations, the total protein level of each homogenized tissue sample was determined using the Bradford protein assay method.

#### 4.4.2. Enzyme-Linked Immunosorbent Assay (ELISA) Procedures

The tissue and serum levels of SF-1 (Cat. No: E3600Ra), IL-6 (Cat. No: E0135Ra), IL-8 (Cat. No: E1167Ra), TNF-α (Cat. No: E0764Ra), VEGF (Cat. No: E0660Ra), HIF-1α (Cat. No: E0210Ra), NF-κB (Cat. No: E0287Ra), and PGE2 (Cat. No: E0504Ra) were quantitatively measured using commercially available rat-specific ELISA kits (Bioassay Technology Laboratory, Shanghai, China). All assay procedures were strictly performed according to the manufacturer’s instructions, without any modifications.

#### 4.4.3. Oxidative Stress Parameters

TAS (Cat. No: E-BC-K801-M) and TOS (Cat. No: E-BC-K802-M) were determined using automated, commercially available colorimetric assay kits (Elabscience Biotechnology, Wuhan, China). OSI was calculated mathematically as the percentage ratio of TOS to TAS.

Absorbance Measurement: Following the addition of the stop solution, the optical density of each well was immediately read at 450 nm using a microplate reader. The final concentrations of the biomarkers in the tissue samples were expressed as pg/mg protein (or ng/mg protein) based on the standard curves generated for each specific kit.

#### 4.4.4. Statistical Analyses

Statistical analyses were conducted utilizing IBM SPSS Statistics software (version 31.0). The Shapiro–Wilk Test was employed to assess whether all continuous variables followed a normal distribution. For those continuous variables that were normally distributed, comparisons among three independent groups were performed using One-Way ANOVA. Subsequent to the variance analysis, pairwise group comparisons were executed using the Bonferroni method, and effect size was assessed via eta squared (η^2^). For continuous variables that did not exhibit a normal distribution, the Kruskal–Wallis Test was applied for comparisons among three independent groups. Pairwise comparisons were conducted using the Dunn Test with Bonferroni Correction, and effect size (r) was calculated using Z values. Continuous variables were reported as mean ± standard deviation, median (minimum-maximum). In graphical representations, bar charts depicting the mean ± standard deviation (mean ± SD) values for each group were utilized, with individual data points for each rat included to illustrate the distribution. Statistical differences between groups were denoted on the graph with connecting lines and corresponding *p*-values. A *p*-value of <0.05 was considered indicative of statistical significance.

## 5. Conclusions

This experimental study has demonstrated that terazosin, an α-1 adrenergic receptor antagonist, effectively suppresses the fundamental pathways involved in the pathogenesis of endometriosis, including SF-1-mediated local steroidogenesis, inflammation, oxidative stress, and angiogenesis. Terazosin, which has been utilized for many years in the treatment of benign prostatic hyperplasia and essential hypertension without a significant side effect profile, presents a potential safe and non-hormonal therapeutic alternative. This is particularly relevant in cases where the use of combined oral contraceptives or progestins is unsuitable due to a desire for pregnancy, in instances where there is no response to current treatments due to progestin resistance, or among patient groups who cannot tolerate GnRH analogs that induce severe hypoestrogenic side effects with prolonged use. To validate these promising preclinical findings and facilitate the integration of this drug into gynecological practice, randomized controlled clinical trials involving actual endometriosis patient populations are necessary.

## Figures and Tables

**Table 1:** Comparison of tissue oxidative stress and inflammatory markers by group *.

Variables	S (*n* = 10) Mean ± SD	E (*n* = 9) Mean ± SD	E + T (*n* = 9) Mean ± SD	E + L (*n* = 10) Mean ± SD	Effect Size (r) **	*p*
TAS (mmol Trolox equiv./L)	2042.14 ± 50.23	538.69 ± 68.81 ^A^	1695.38 ± 107.50 ^B^	1692.46 ± 170.44 ^B^	0.04	<0.001
TOS (µmol H_2_O_2_ equiv./L)	39.60 ± 5.75	85.55 ± 3.98 ^A^	40.39 ± 2.61 ^B^	42.87 ± 5.88 ^B^	0.18	<0.001
OSI (TOS/TASX100)	1.94 ± 0.29	16.10 ± 2.08 ^A^	2.39 ± 0.26 ^B^	2.58 ± 0.52 ^B^	0.06	<0.001
VEGF (pg/mg protein)	93.13 ± 16.98	354.76 ± 46.42 ^A^	125.99 ± 27.07 ^B^	120.31 ± 12.37 ^B^	0.04	<0.001
TNF-α (pg/mg protein)	87.77 ± 14.10	352.60 ± 39.78 ^A^	165.45 ± 42.62 ^B^	160.43 ± 26.20 ^B^	0.03	<0.001
IL-8 (pg/mg protein)	43.23 ± 9.06	175.38 ± 14.13 ^A^	77.42 ± 17.31 ^B^	76.56 ± 11.77 ^B^	0.02	<0.001
NF-κB (pg/mg protein)	455.28 ± 109.38	2163.81 ± 243.66 ^A^	705.38 ± 165.99 ^B^	703.64 ± 74.98 ^B^	0.02	<0.001
IL-6 (pg/mg protein)	1.44 ± 0.53	6.43 ± 0.86 ^A^	3.60 ± 1.04 ^B^	4.17 ± 0.54 ^B^	0.16	<0.001
PGE2 (pg/mg protein)	491.73 ± 125.42	1548.84 ± 261.43 ^A^	877.44 ± 171.11 ^B^	818.99 ± 100.42 ^B^	0.14	<0.001
HIF-1α (pg/mg protein)	914.52 ± 150.72	3538.07 ± 421.06 ^A^	1366.43 ± 298.97 ^B^	1409.34 ± 216.46 ^B^	0.07	<0.001
SF-1 (pg/mg protein)	15,066.28 ± 2791.52	60,789.32 ± 8618.61 ^A^	20,599.26 ± 5101.25 ^B^	20,880.14 ± 2540.16 ^B^	0.10	<0.001

S: Sham; E: Endometriosis; E + T: Endometriosis + Terazosin; E + L: Endometriosis + Leuprolide; * different letters (A, B) in the same row indicate a statistically significant difference (*p* < 0.05), ** effect size between E + T and E + L groups.

**Table 2:** Comparison of serum oxidative stress and inflammatory markers by group *.

Variables	S (*n* = 10) Mean ± SD	E (*n* = 9) Mean ± SD	E + T (*n* = 9) Mean ± SD	E + L (*n* = 10) Mean ± SD	Effect Size (r) **	*p*
TAS (mmol Trolox equiv./L)	1465.75 ± 81.21	657.14 ± 123.82 ^A^	1330.60 ± 126.18 ^B^	1268.26 ± 88.39 ^B^	0.15	<0.001
TOS (µmol H_2_O_2_ equiv./L)	32.95 ± 4.65	60.00 ± 3.98 ^A^	36.10 ± 2.81 ^B^	39.69 ± 7.28 ^B^	0.10	<0.001
OSI (TOS/TASX100)	2.25 ± 0.32	9.43 ± 1.83 ^A^	2.73 ± 0.37 ^B^	3.17 ± 0.62 ^B^	0.21	<0.001
VEGF (pg/mg protein)	302.54 ± 45.81	576.61± 61.88 ^A^	343.60 ± 45.31 ^B^	377.25 ± 42.17 ^B^	0.24	<0.001
TNF-α (pg/mg protein)	243.55 ± 25.95	523.70 ± 42.65 ^A^	367.42 ± 32.26 ^B^	364.72 ± 31.26 ^B^	0.03	<0.001
IL-8 (pg/mg protein)	99.96 ± 8.92	213.21 ± 18.07 ^A^	127.23 ± 18.60 ^B^	145.98 ± 11.03 ^B^	0.32	<0.001
NF-κB (pg/mg protein)	2120.80 ± 389.58	2441.33 ± 292.81	2248.66 ± 433.02	2420.40 ± 359.61	0.30	0.206
IL-6 (pg/mg protein)	5.50 ± 0.68	8.92 ± 0.91 ^A^	6.69 ± 0.50 ^B^	6.71 ± 0.63 ^B^	0.01	<0.001
PGE2 (pg/mg protein)	1340.70 ± 199.73	2096.33 ± 148.03 ^A^	1504.55 ± 126.99 ^B^	1612.40 ± 183.62 ^B^	0.22	<0.001
HIF-1α (pg/mg protein)	2689.89 ± 495.56	4010.50 ± 616.06 ^A^	2792.65 ± 383.44 ^B^	2977.04 ± 523.61 ^B^	0.14	<0.001
SF-1 (pg/mg protein)	73,966.60 ± 6166.26	105,200.66 ± 7423.26 ^A^	82,781.11 ± 7992.15 ^B^	91,657.14 ± 11,621.95 ^B^	0.22	<0.001

S: Sham; E: Endometriosis; E + T: Endometriosis + Terazosin; E + L: Endometriosis + Leuprolide; * different letters (A, B) in the same row indicate a statistically significant difference (*p* < 0.05); ** effect size between E + T and E + L groups.

**Table 3:** Comparison of peritoneal fluid oxidative stress and inflammatory markers by group *.

Variables	S (*n* = 10) Mean ± SD	E (*n* = 9) Mean ± SD	E + T (*n* = 9) Mean ± SD	E + L (*n* = 10) Mean ± SD	Effect Size (r) **	*p*
TAS (mmol Trolox equiv./L)	1359.74 ± 124.39	301.72 ± 72.00 ^A^	695.45 ± 101.82 ^B^	721.17 ± 92.19 ^B^	0.09	<0.001
TOS (µmol H_2_O_2_ equiv./L)	32.59 ± 8.13	70.34 ± 4.37 ^A^	46.57 ± 5.05 ^B^	55.30 ± 6.25 ^B^	0.33	<0.001
OSI (TOS/TASX100)	2.44 ± 0.78	24.59 ± 6.17 ^A^	6.89 ± 1.6 ^B^	7.59 ± 0.88 ^B^	0.18	<0.001
VEGF (pg/mg protein)	129.09 ± 23.94	312.05 ± 53.93 ^A^	141.66 ± 22.89 ^B^	155.31 ± 27.60 ^B^	0.19	<0.001
TNF-α (pg/mg protein)	125.28 ± 22.33	303.72 ± 64.88 ^A^	190.24 ± 15.37 ^B^	181.10 ± 34.14 ^B^	0.09	<0.001
IL-8 (pg/mg protein)	55.74 ± 9.76	140.78 ± 23.56 ^A^	74.84 ± 9.70 ^B^	74.48 ± 11.40 ^B^	0.01	<0.001
NF-κB (pg/mg protein)	930.64 ± 131.47	1652.10 ± 234.35 ^A^	977.72 ± 120.13 ^B^	993.01 ± 129.75 ^B^	0.08	<0.001
IL-6 (pg/mg protein)	1.76 ± 0.22	4.44 ± 0.65 ^A^	2.65 ± 0.16 ^B^	2.49 ± 0.37 ^B^	0.12	<0.001
PGE2 (pg/mg protein)	691.27 ± 92.37	1447.30 ± 146.16 ^A^	909.46 ± 54.79 ^B^	954.88 ± 156.80 ^B^	0.09	<0.001
HIF-1α (pg/mg protein)	1359.74 ± 124.39	301.72 ± 72.00 ^A^	695.45 ± 101.82 ^B^	721.17 ± 92.19 ^B^	0.07	<0.001
SF-1 (pg/mg protein)	32.59 ± 8.13	70.34 ± 4.37 ^A^	46.57 ± 5.05 ^B^	55.30 ± 6.25 ^B^	0.13	<0.001

S: Sham; E: Endometriosis; E + T: Endometriosis + Terazosin; E + L: Endometriosis + Leuprolide; * different letters (A, B) in the same row indicate a statistically significant difference (*p* < 0.05); ** effect size between E + T and E + L groups.

**Table 4:** Distribution of histopathological scores across the experimental groups.

Group	*n*	Score 0 *n* (%)	Score 1 *n* (%)	Score 2 *n* (%)	Score3 *n* (%)	Median (min–max)
S	10	10 (100.0)	0 (0.0)	0 (0.0)	0 (0.0)	0 (0–0) ^A^
E	9	0 (0.0)	0 (0.0)	3 (33.3)	6 (66.7)	3 (2–3) ^B^
E + T	9	3 (33.3)	3 (33.3)	3 (33.3)	0 (0.0)	1 (0–2) ^A^
E + L	10	4 (40.0)	4 (40.0)	2 (20.0)	0 (0.0)	1 (0–2) ^A^

S: Sham; E: Endometriosis; E + T: Endometriosis + Terazosin; E + L: Endometriosis + Leuprolide; Different letters (A, B) in the same row indicate a statistically significant difference (*p* < 0.05).

## Data Availability

The raw data supporting the findings of this study have been deposited in the Mendeley Data repository and are publicly accessible at: doi: 10.17632/c2ybb3kzdf.1.
